# STAT1-Dependent Recruitment of Ly6C^hi^CCR2^+^ Inflammatory Monocytes and M2 Macrophages in a Helminth Infection

**DOI:** 10.3390/pathogens10101287

**Published:** 2021-10-06

**Authors:** Mireya Becerra-Díaz, Yadira Ledesma-Soto, Jonadab E. Olguín, Angel Sánchez-Barrera, Mónica G. Mendoza-Rodríguez, Sandy Reyes, Abhay R. Satoskar, Luis I. Terrazas

**Affiliations:** 1 Unidad de Biomedicina, Facultad de Estudios Superiores (FES)-Iztacala, Universidad Nacional Autónoma de Mexico (UNAM), Av. De Los Barrios, Los Reyes Iztacala, Tlalnepantla 54090, Edo. de Mexico, Mexico; mbecerr1@jhmi.edu (M.B.-D.); yadira.ledesma@unam.mx (Y.L.-S.); je.olguin@iztacala.unam.mx (J.E.O.); dragon_puma@comunidad.unam.mx (A.S.-B.); monica.mendoza@iztacala.unam.mx (M.G.M.-R.); sandy_reyes25@comunidad.unam.mx (S.R.); 2 Department of Anesthesiology and Critical Care Medicine, Johns Hopkins University School of Medicine, Baltimore, MD 21205, USA; 3 Laboratorio Nacional en Salud FES-Iztacala, Universidad Nacional Autónoma de Mexico (UNAM), Av. De Los Barrios 1, Los Reyes Iztacala, Tlalnepantla 54090, Edo. de Mexico, Mexico; 4 Department of Pathology, The Ohio State University, Columbus, OH 43210, USA; Abhay.Satoskar@osumc.edu

**Keywords:** helminth, STAT1, M2 macrophages, inflammatory monocytes, Taenia

## Abstract

Signal Transducer and Activator of Transcription (STAT) 1 signaling is critical for IFN-γ-mediated immune responses and resistance to protozoan and viral infections. However, its role in immunoregulation during helminth parasitic infections is not fully understood. Here, we used STAT1^−/−^ mice to investigate the role of this transcription factor during a helminth infection caused by the cestode *Taenia crassiceps* and show that STAT1 is a central molecule favoring susceptibility to this infection. STAT1^−/−^ mice displayed lower parasite burdens at 8 weeks post-infection compared to STAT1^+/+^ mice. STAT1 mediated the recruitment of inflammatory monocytes and the development of alternatively activated macrophages (M2) at the site of infection. The absence of STAT1 prevented the recruitment of CD11b^+^Ly6C^hi^Ly6G^−^ monocytic cells and therefore their suppressive activity. This failure was associated with the defective expression of CCR2 on CD11b^+^Ly6C^hi^Ly6G^−^ cells. Importantly, CD11b^+^Ly6C^hi^Ly6G^−^ cells highly expressed PDL-1 and suppressed T-cell proliferation elicited by anti-CD3 stimulation. PDL-1^+^ cells were mostly absent in STAT1^−/−^ mice. Furthermore, only STAT1^+/+^ mice developed M2 macrophages at 8 weeks post-infection, although macrophages from both *T. crassiceps*-infected STAT1^+/+^ and STAT1^−/−^ mice responded to IL-4 in vitro, and both groups of mice were able to produce the Th2 cytokine IL-13. This suggests that CD11b^+^CCR2^+^Ly6C^hi^Ly6G^−^ cells give rise to M2 macrophages in this infection. In summary, a lack of STAT1 resulted in impaired recruitment of CD11b^+^CCR2^+^Ly6C^hi^Ly6G^−^ cells, failure to develop M2 macrophages, and increased resistance against *T. crassiceps* infection.

## 1. Introduction

Signal Transducers and Activators of Transcription (STATs) are latent cytoplasmic transcription factors that strongly participate in the regulation of the immune system as well as in diverse processes such as cellular proliferation, anti-apoptosis, and angiogenesis, among others [1,2]. STAT1 was the first member discovered in this family of proteins, and it is well-known for its participation in the innate and adaptive immune response. Additionally, STAT1 has been largely associated with resistance against protozoan parasites and bacterial and viral infections [1,3].

During intracellular parasitic infections such as those caused by *Leishmania mexicana*, *Trypanosoma cruzi*, and *Toxoplasma gondii,* STAT1 leads to cellular Th1 responses through proinflammatory cytokines such as gamma interferon (IFN-γ) and M1 macrophage polarization, which have been associated with resistance to these infections [4,5,6]. In contrast, STAT1^−/−^ mice fail to mount a significant Th1 response and cannot control these protozoan infections [4,7,8], indicating that the STAT1-mediated signaling pathway favors the development of protective immunity by inhibiting Th2-type responses. In other parasitic infections, such as those caused by helminths, the role of STAT1 is still not completely understood.

*Taenia crassiceps* is a helminth tapeworm that belongs to the Class Cestoda, which generates natural infections in some definitive canine hosts, such as dogs, red foxes, and wolves. Its larval phase, called metacestode or cysticerci, has an extensive asexual reproduction rate in the pleural and peritoneal cavities of their intermediate hosts, such as wild rodents [9]. *T. crassiceps* is commonly used as a mouse model to study cysticercosis [9]. During acute infection by this helminth, a strong Th1 immune response characterized by high levels of IL-2 and IFN-γ is induced and has been associated with host protection [10], but as the infection progresses, levels of both IL-2 and IFN-γ decrease as well as IL-12 produced by macrophages [11]. These reduced levels of Th1-type cytokines correlate with increased levels of IL-4 at chronic infection stages, suggesting a switch from an inflammatory response in the acute infection toward an anti-inflammatory response in the chronic infection, favoring a permissive microenvironment where the parasite can develop and persist in the host. Susceptibility to *T. crassiceps* infection is STAT6-mediated [11], whereas resistance to this infection is STAT4-dependent [12]. Therefore, unlike most helminth parasitic infections, protection during experimental cysticercosis is mediated by Th1-type immune responses, while parasite establishment is associated with Th2-type mediated immune responses [13]. Additionally, experimental cysticercosis caused by *T. crassiceps* reduces the proliferative capacity of T cells obtained from infected mice to either non-specific or specific stimulation, suggesting the strong immunosuppressive capacity of this helminth [13]. 

The purpose of this study was to evaluate the role of STAT1-mediated signaling in determining the outcome of experimental murine cysticercosis by the cestode *T. crassiceps*. To approach this question, we compared the course of *T. crassiceps* infection in STAT1^−/−^ BALB/c mice (STAT1^−/−^) to that of wild-type BALB/c (STAT1^+/+^) mice. In addition, we analyzed both the antibody and cytokine profiles, as well as the phenotype of acutely recruited peritoneal cells and macrophages at the site of infection. Our data demonstrate that the STAT1 signaling pathway has a critical role in recruiting inflammatory monocytes that become M2 macrophages and favors experimental cysticercosis.

## 2. Results

### 2.1. Lack of STAT1 Leads to a Reduction in Susceptibility to Experimental Cysticercosis Caused by T. crassiceps

**Figure 1:**
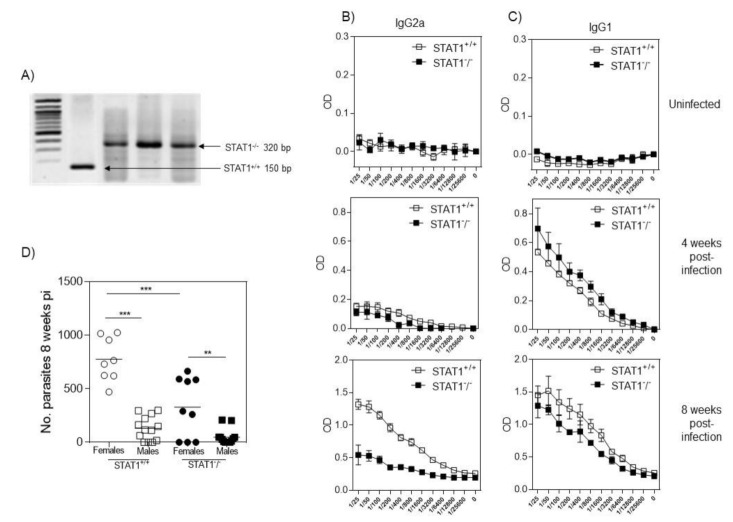
Antibody profile and parasite burden in response to *T. crassiceps* infection in STAT1^+/+^ and STAT1^−/−^ mice. (**A**) Before infection, all mice were genotyped for the absence of STAT1. After infection, serum from uninfected and infected mice (4 and 8 weeks post-infection) was obtained by centrifugation of peripheral blood. (**B**) Serum analysis of specific IgG2a and (**C**) specific IgG1 antibodies against *T. crassiceps* soluble antigen was performed by ELISA. (**D**) Parasite burden at 8 weeks p.i. in female and male STAT1^+/+^ and STAT1^−/−^ mice. STAT1^−/−^ mice displayed significantly lower parasite burdens than STAT1^+/+^ mice, despite a decreased Th1-type antibody response. Mice were i.p. infected with 10 metacestodes of *T. crassiceps* and, 8 weeks later, the parasite burden was recorded. The data in the graphs are combined from three independent experiments. n = 8 mice per group; * *p* < 0.05; ** *p* < 0.01.

In order to analyze the role of STAT1 in the immunoregulation of a helminth infection, we used a mouse model of cysticercosis caused by the cestode *T. crassiceps*. First, we confirmed the absence of the STAT1 gene by genotyping-PCR in our knockout mice (Figure 1A). Following *T. crassiceps* i.p. infection with 10 cysticerci, we determined the antibody production against *T. crassiceps* in the serum of STAT1^+/+^ and STAT1^−/−^ mice obtained at weeks 4 and 8 post-infection to detect specific anti-*T. crassiceps* IgG1 and IgG2a antibodies. STAT1^+/+^ mice displayed a significantly higher titer of IgG2a anti-*T. crassiceps* antibodies than STAT1^−/−^ at 4 and 8 weeks post-infection (Figure 1B), whereas there were no significant differences in IgG1 production between STAT1^+/+^ and STAT1^−/−^ mice at any time during infection (Figure 1C). 

At week 8 after infection, mice were euthanized, and the parasitic burdens were registered. We found, as previously reported, that female mice were more susceptible to *T. crassiceps* infection by around 75% in comparison to males [14]. *T. crassiceps*-infected STAT1^+/+^ female mice harbored higher numbers of parasites compared to STAT1^+/+^ males (Figure 1D). Interestingly, the absence of STAT1 resulted in a significant reduction in the parasitic burden in both male and female mice compared to STAT1^+/+^ mice (Figure 1D). A reduction of ~60% in the parasitic burden was observed in STAT1^−/−^ female mice compared to their wild-type counterparts, whereas STAT1^−/−^ males almost cleared the infection and harbored a very low number of parasites (Figure 1D); however, when compared to STAT1^+/+^ male mice, no statistical differences were observed.

These data suggest that STAT1 expression could be related to the establishment of a helminth parasite such as *T. crassiceps.*

### 2.2. Dysregulation of Monocyte Recruitment during Early T. crassiceps Infection in STAT1^−/−^ Mice

**Figure 2:**
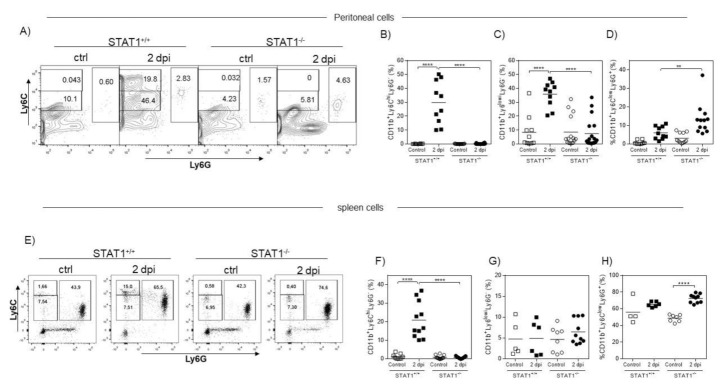
Mice lacking STAT1 display impaired Ly6C^+^ and exacerbated Ly6G^+^ cell recruitment in acute *T. crassiceps* infection. Flow cytometry was performed to obtain peritoneal exudate containing cells and splenocytes from uninfected and acute (2 days) *T. crassiceps* infected STAT1^+/+^ and STAT1^−/−^ mice. All analyses were done after gating CD11b^+^ cells. (**A**) Representative dot plot showing the expression of Ly6C and Ly6G in PECs from STAT1^+/+^ and STAT1^−/−^ mice. (**B**) Graphs showing the percentage of CD11b^+^Ly6C^hi^Ly6G^−^ cells, (**C**) CD11b^+^Ly6C^low^Ly6G^−^ cells, and (**D**) CD11b^+^Ly6C^low^Ly6G^+^ cells in peritoneal exudate from STAT1^+/+^ and STAT1^−/−^ mice. (**E**) Representative dot plot showing the expression of Ly6C and Ly6G in splenocytes from STAT1^+/+^ and STAT1^−/−^ mice. (**F**) Graphs showing the percentage of CD11b^+^Ly6C^hi^Ly6G^−^ cells, (**G**) CD11b^+^Ly6C^low^Ly6G^−^ cells, and (**H**) CD11b^+^Ly6C^low^Ly6G^+^ cells in splenocytes from STAT1^+/+^ and STAT1^−/−^ mice. The data in the graphs are representative of three independent experiments and are shown as the mean percentage change in expression ± S.E. n = 6–10 mice per group; * *p* < 0.05; ** *p* < 0.01; *** *p* < 0.001.

Henceforth, all the other measurements refer only to females, because, in this genus, the greatest differences in susceptibility to the parasite were observed. A first step in innate immunity is the fast recruitment of immune cells to the site of infection, with the purpose of clearing the pathogen [15]. Monocytes and neutrophils are cell populations that rapidly arrive at infected or damaged tissues [16]. Thus, to investigate the early response at the site of infection with *T. crassiceps*, we analyzed the monocyte and neutrophil recruitment as early as 2 days after infection in the peritoneal cavity and in the spleen. Inflammatory monocytes were identified as CD11b^+^LyC6^hi^Ly6G^−^ and neutrophils as CD11b^+^Ly6^low^Ly6G^+^ by flow cytometry. Two days after *T. crassiceps* infection, peritoneal exudate cells (PECs) were obtained and processed as indicated in the Materials and Methods. The rapid mobilization or recruitment of inflammatory monocytes CD11b^+^LyC6^hi^Ly6G^−^ was observed in the peritoneal cavity of STAT1^+/+^ mice (Figure 2A), reaching up to 50% of all CD11b^+^ cells. In clear contrast, infected STAT1^−/−^ mice were unable to recruit this population in response to *T. crassiceps* (Figure 2B). Similarly, a subpopulation identified as CD11b^+^Ly6C^low^Ly6G^−^ was recruited below 40% of CD11b^+^ cells only in STAT1^+/+^ mice (Figure 2C), while STAT1^−/−^ mice failed to recruit this population. Then, the recruitment of neutrophils (CD11b^+^Ly6C^−^Ly6G^+^) was analyzed. We found a significant increase in the recruitment of CD11b^+^Ly6C^−^Ly6G^+^ cells in STAT1^−/−^ infected mice compared to STAT1^+/+^ mice (Figure 2D). 

To determine whether these differences in the recruitment of inflammatory cells at the site of infection were also reflected in the lymphoid organs, such as the spleen, we analyzed the same cell populations in the spleen as early as 2 days after *T. crassiceps* infection. High percentages of inflammatory monocytes (CD11b^+^LyC6^hi^Ly6G^−^) were detected in the spleens of STAT1^+/+^ mice infected with *T. crassiceps* (Figure 2E–H). In contrast, similarly infected STAT1^−/−^ mice displayed a remarkable inability to recruit this cell population but maintained the ability to recruit the CD11b^+^Ly6C^low^Ly6G^+^ population (Figure 2E–H). These data suggest an early dysregulation in the recruitment of innate immune cells during experimental cysticercosis in the absence of STAT1. 

**Figure 3:**
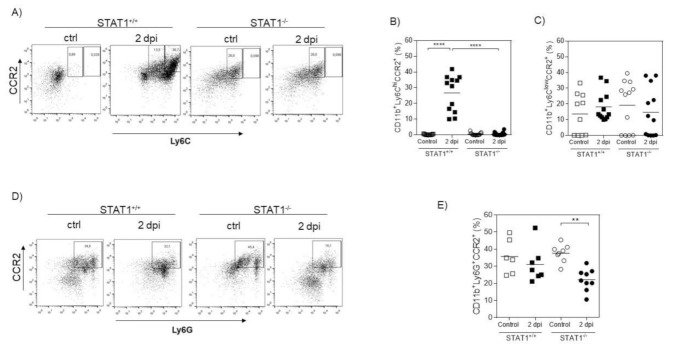
The absence of STAT1 impairs the expression of CCR2 in monocytes. Flow cytometry was performed on peritoneal exudate cells and splenocytes from uninfected and acute (2 days) *T. crassiceps*-infected STAT1^+/+^ and STAT1^−/−^ mice. All analyses were done after gating CD11b^+^ cells. (**A**) Representative dot plot showing the expression of CCR2 on Ly6C in PECs from STAT1^+/+^ and STAT1^−/−^ mice. (**B**) Graphs showing the percentage of CD11b^+^Ly6C^hi^CCR2^+^Ly6G^−^ cells. (**C**) CD11b^+^Ly6C^low^CCR2^+^Ly6G^−^ cells. (**D**) Representative dot plot showing the expression of CCR2 on Ly6G in PECs from STAT1^+/+^ and STAT1^−/−^ mice. (**E**) CD11b^+^Ly6C^low^CCR2^+^Ly6G^+^ cells in peritoneal exudate from STAT1^+/+^ and STAT1^−/−^ *T. crassiceps*-infected mice. The data in the graph are representative of three independent experiments and are shown as the mean percentage change in expression ± S.E. n = 6–9 mice; * *p* < 0.05; ** *p* < 0.01; **** *p* < 0.001.

In order to reveal a possible mechanism involved in the failure to recruit innate immune cells in the absence of STAT1 at 2 days post-infection, we analyzed the expression of CCR2, a chemokine receptor associated with the recruitment of inflammatory monocytes [17,18]. Data shown in Figure 3A–D indicate that STAT1^−/−^ *T. crassiceps*-infected mice were unable to recruit inflammatory monocytes expressing CCR2. In contrast, STAT1^+/+^ mice recruited up to 40% of CCR2-expressing monocytes.

### 2.3. Monocytic Cells Recruited in STAT1^+/+^ Mice Express PDL-1

**Figure 4:**
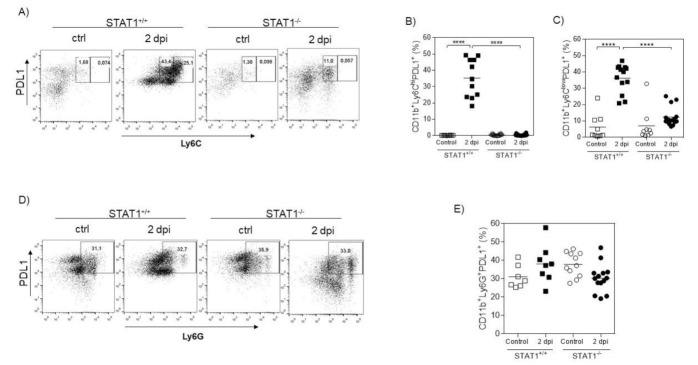
Monocytic Ly6C^+^ cells from STAT1^−/−^ mice i.p. infected with *T. crassiceps* fail to express PDL-1. Peritoneal exudate cells from female STAT1^+/+^ and STAT1^−/−^ mice were obtained from uninfected and infected mice 2 days post-infection. Membrane molecule expression was analyzed by FACS. (**A**) Analysis of PDL-1 and Ly6C in CD11b^+^ gated cells from naïve and infected STAT1^+/+^ and STAT1^−/−^ mice 2 days post-infection. (**B**) Analysis of PDL-1 and Ly6G in CD11b^+^ gated cells from naïve and infected STAT1^+/+^ and STAT1^−/−^ mice 2 days post-infection. Graphs showing the percentages of (**C**) CD11b^+^Ly6C^hi^PDL-1^+^ cells, (**D**) CD11b^+^Ly6C^lo^PDL-1^+^ cells, and (**E**) CD11b^+^Ly6G^+^PDL-1^+^ are provided. The data in the graphs are combined from two independent experiments and are shown as the mean percentage change in expression ± S.E. n = 6 mice per group; * *p* < 0.05; ** *p* < 0.01; **** *p* < 0.001.

Next, we analyzed the expression of PDL-1, a molecule associated with the regulation of T-cell proliferation [19]. Using a flow cytometric approach, we found that *T. crassiceps* infection promoted the recruitment of CD11b^+^LyC6^hi^Ly6G^−^ and CD11b^+^Ly6C^low^Ly6G^−^ monocytic cells expressing PDL-1 in STAT1^+/+^ mice, whereas similarly infected STAT1^−/−^ mice were unable to significantly recruit this population (Figure 4A–C). No differences in the expression of PDL-1 were observed among PECs of STAT1^+/+^ and STAT1^−/−^ mice in CD11b^+^Ly6C^−^Ly6G^+^ neutrophilic cells (Figure 4D,E). 

### 2.4. STAT1 Is Necessary for the Anti-Proliferative Activity of CD11b^+^LyC6^hi^Ly6G^−^ Monocytic Cells Recruited during Early Infection with T. crassiceps

**Figure 5:**
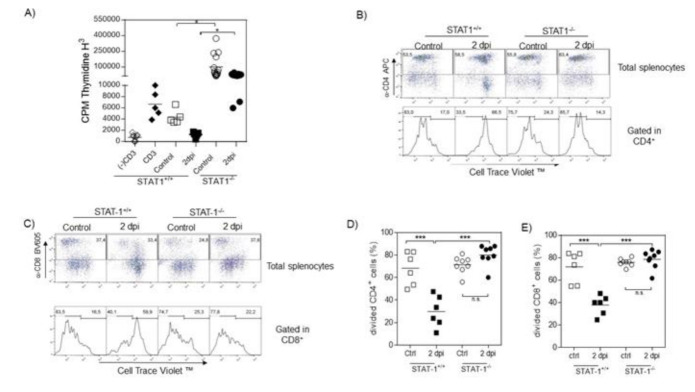
Peritoneal exudate cells obtained 2 days after infection from STAT1^+/+^ mice are able to suppress T-cell proliferation. Peritoneal exudate cells from female STAT1^+/+^ and STAT1^−/−^ mice were obtained from uninfected and infected mice 2 days post-infection that were co-cultured with previously anti-CD3 (CD3) stimulated splenocytes at 1:4 ratio for 72 h or left without stimuli (-CD3), also called controls (Ctrl). (**A**) [^3^H]-labelled thymidine uptake. (**B**,**C**) Dot plots and histograms of splenocytes from naïve STAT1^+/+^ mice stained with Cell Trace Violet™ and stimulated with anti-CD3 antibody; then, peritoneal cells from naïve or *T. crassiceps*-infected STAT1^+/+^ or STAT1^−/−^ female mice 2 days post-infection were added to a final 1:4 dilution in a humidified atmosphere containing 5% CO_2_ at 37 °C. After 72 hours of incubation, cells were harvested, stained for CD4 and CD8 molecules, and immediately analyzed by flow cytometry. (**D**,**E**) Percentages of CD4^+^ cells and CD8^+^ cells divided, respectively. Each square or circle indicates an individual mouse; * *p* < 0.05; ** *p* < 0.01; *** *p* < 0.001.

Several reports have indicated the suppressive potential of monocytes and immature neutrophils in different parasitic diseases [20,21,22]. To evaluate such activity in the presence and absence of STAT1, we isolated PECs from STAT1^+/+^ and STAT1^−/−^ *T. crassiceps*-infected (2 days after infection) or naïve mice. Peritoneal exudate cells were co-cultured with splenocytes from naive STAT1^+/+^ mice previously stimulated with plate-bound coated anti-CD3 antibody and cell proliferation was evaluated using ^3^HTDR uptake. As observed in Figure 5A, co-cultures of PECs from STAT1^+/+^ mice recruited during infection with *T. crassiceps* significantly suppressed ^3^HTDR uptake by total splenocytes. In contrast, PECs from *T. crassiceps*-infected STAT1^−/−^ mice did not. To further determine whether the suppressed cell proliferation was restricted to CD4 or CD8 cells, we used the Cell Trace Violet technique. PECs from STAT1^+/+^ mice recruited acutely during infection with this helminth significantly inhibited the proliferative response of both CD4^+^ and CD8^+^ splenocytes, reducing this response by at least 50% (Figure 5B–E). Remarkably, PECs from STAT1^−/−^ infected mice were unable to suppress the proliferative response of either CD4^+^ or CD8^+^ splenocytes (Figure 5B–E). 

### 2.5. Peritoneal Macrophages from STAT1^−/−^ T. crassiceps-Infected Mice Fail to Express Characteristic M2-Genes but Are Able to Respond to IL-4 In Vitro

**Figure 6:**
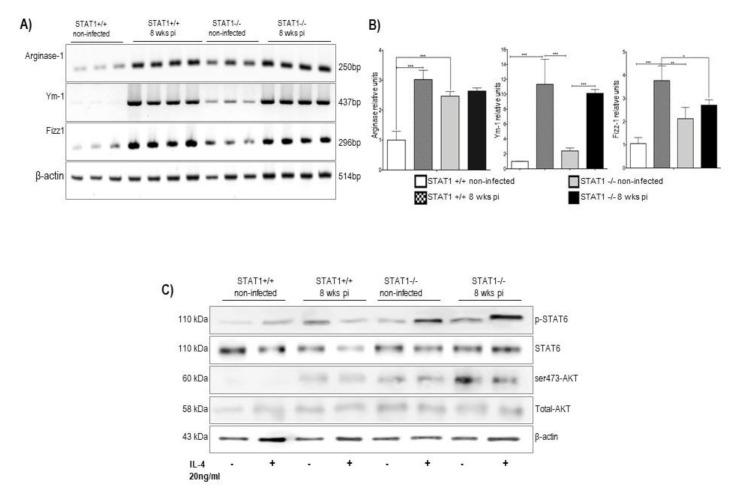
Peritoneal macrophages from STAT1^−/−^ mice infected with *T. crassiceps* show impaired expression of genes associated with an M2 phenotype but normal response to IL-4. Peritoneal exudate cells from female STAT1^+/+^ and STAT1^−/−^ mice were obtained, and macrophage populations were enriched by adherence. Macrophages were processed for RNA extraction. The RNA was quantified and reverse-transcribed. Once cDNA was obtained, conventional PCR was performed, as described in Materials and Methods. (**A**) RT-PCR products for Arginase-1, Ym1, Fizz1 and β-Actin from STAT1^+/+^ and STAT1^−/−^ uninfected and infected mice 8 weeks post-infection. (**B**) Graphic analyses of the relative expression of Arginase 1, Ym 1, and Fizz 1 genes, normalized to β-Actin expression. (**C**) Western blot assays of macrophages from naive or *T. crassiceps*-infected mice in response to IL-4. Macrophages were obtained from the peritoneal cavity, enriched by adherence, stimulated with recombinant murine IL-4 (20 ng/mL) for 30 min, and analyzed for the phosphorylation of STAT6 and AKT; β-actin was used as a load protein control. * *p* < 0.05; ** *p* < 0.01; *** *p* < 0.001. For A and B, the data in the graphs are combined from two independent experiments and are shown as the mean percentage change in expression ± S.E. n = 4 mice per group; * *p* < 0.05; ** *p* < 0.01.

Along with Th2-polarized responses, most helminth infections promote the induction of M2 macrophages, which can be beneficial for either the host [23,24,25] or the parasite [13,21,26]. *T. crassiceps* infection is known to induce large amounts of M2 macrophages in chronic stages [13]. Thus, we determined how a lack of STAT1 may alter macrophage polarization. We evaluated the expression of characteristic M2 genes by RT-PCR. Peritoneal macrophages from STAT1^+/+^ and STAT1^−/−^ recruited at 8 weeks post-infection were analyzed for the expression of *Arginase-1*, *Fizz1*, and *Ym1*. Macrophages from infected STAT1^+/+^ mice displayed robust and significant expression of *Arginase-1*, *Fizz1*, and *Ym1* compared to macrophages from STAT1^+/+^ naïve mice (Figure 6A,B). In clear contrast, peritoneal macrophages from *T. crassiceps*-infected STAT1^−/−^ mice displayed limited expression of such genes (Figure 6A,B). Only the expression of Ym1 was induced in STAT1^−/−^ infected mice (Figure 6B). 

 Considering that the M2 macrophage phenotype is mainly driven by IL-4, we looked for some defective signaling responses to this cytokine in macrophages lacking STAT1. We stimulated peritoneal macrophages obtained from STAT1^−/−^ and STAT1^+/+^ naïve or *T. crassiceps*-infected mice with 20 ng/mL of IL-4 for 30 min. As observed in Figure 6C, STAT1^−/−^ macrophages from naïve or *T. crassiceps*-infected mice showed a potent response to IL-4 stimulation mediated by both STAT6 and AKT phosphorylation. This indicates that a lack of STAT1 did not impair the macrophage response to IL-4. Moreover, macrophages from STAT1^−/−^ *T. crassiceps*-infected mice displayed an enhanced response to IL-4, as demonstrated by higher levels of STAT6 phosphorylation compared to STAT1^+/+^ macrophages. All these data suggest that macrophages lacking STAT1 can respond to M2 stimulation.

### 2.6. Impaired M2 Macrophage Polarization in T. crassiceps-Infected STAT1^−/−^ Mice 

**Figure 7:**
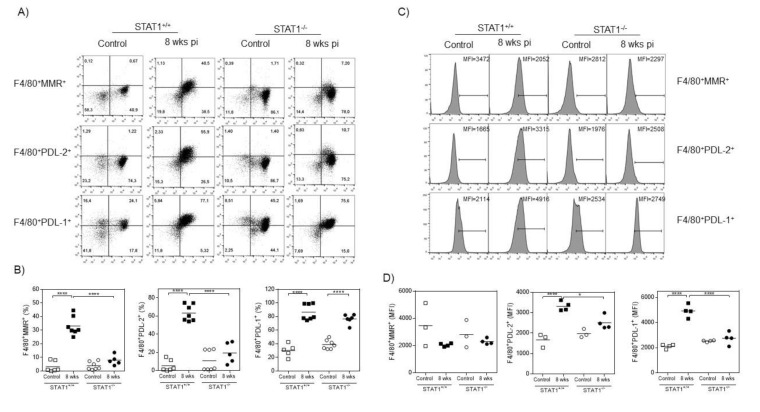
Impaired M2 macrophage polarization in STAT1^−/−^ mice infected with *T. crassiceps.* Peritoneal exudate cells were obtained from STAT1^+/+^ and STAT1^−/−^ naïve or *T. crassiceps*-infected mice 8 weeks post-infection. Cells were stained for F4/80 and for the indicated M2 markers. (**A**) Representative dot plots of macrophages expressing mannose receptor (MMR), PDL-2, and PDL-1. (**B**) Graphic analysis of the surface expression of M2 markers in peritoneal macrophages. (**C**) Histograms displaying median fluorescence intensity (MFI) of the indicated M2 markers in F4/80^+^ peritoneal cells. (**D**) Analysis of MFI of the indicated M2 markers in peritoneal macrophages. The data in the graphs are combined from two independent experiments and are shown as the mean percentage change in expression ± S.E. n = 4–7 mice per group; *p < 0.05; **p < 0.01; ***p < 0.001.

Next, we analyzed by flow cytometry the surface markers (MMR, PDL-1, and PDL-2) associated with the M2 macrophage phenotype in peritoneal macrophages recruited at 8 weeks after *T. crassiceps* infection from STAT1^+/+^ and STAT1^−/−^ mice (Figure 7). The expression of these molecules was evaluated as the percentage of positive cells and as the mean fluorescence intensity (MFI). Macrophages from STAT1^+/+^ mice displayed higher percentages of MMR expression as well as higher MFI (Figure 7A–D). Similarly, peritoneal macrophages from STAT1^+/+^ infected mice displayed a higher percentage of cells expressing both PDL-1 and PDL-2, and enhanced MFI for these membrane molecules (Figure 7A–D). By contrast, macrophages from STAT1^−/−^ *T. crassiceps*-infected mice expressed minimal levels of MMR and PDL-2, critical markers associated with M2 macrophage polarization (Figure 7A–C). Interestingly, STAT1^−/−^ mice developed PDL-1^+^ macrophages at 8 weeks post-infection, but no enhancement in PDL-1 MFI was found compared to STAT1^−/−^ naïve mice (Figure 7A–D). 

### 2.7. A Similar Th2 Cytokine Profile Is Observed in Sera from Both STAT1^+/+^ and STAT1^−/−^ Mice Infected with T. crassiceps

**Figure 8:**
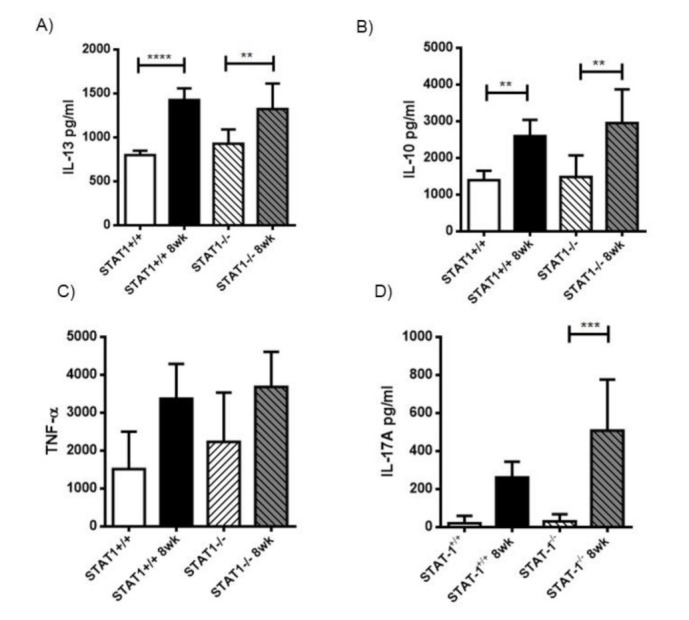
Global cytokine profile in STAT1^−/−^ mice infected with *T. crassiceps*. Sera were obtained from STAT1^+/+^ and STAT1^−/−^ naïve or *T. crassiceps*-infected mice 8 weeks post-infection, and different cytokines were evaluated by ELISA. (**A**) IL-13 levels. (**B**) IL-10 levels. (**C**) TNF-a levels and (**D**) IL-17A. The data in the graphs are combined from two independent experiments and are shown as the mean percentage change in expression ± S.E. n = 6 mice per group; * *p* < 0.05; ** *p* < 0.01; *** *p* < 0.001.

The impaired M2 macrophage profile observed in STAT1^−/−^ mice may be a consequence of failed Th2 cytokine production. Thus, we obtained sera from infected STAT1^−/−^ mice 8 weeks post-infection and compared their cytokine levels to equally infected STAT1^+/+^ mice. Concentrations of IL-13, IL-10, TNF-α, and IL-17 in sera were measured by ELISA. *T. crassiceps*-infected STAT1^−/−^ and STAT1^+/+^ mice had increased levels of IL-13 and IL-10, compared to uninfected mice. No differences in IL-13 and IL-10 levels were found between infected STAT1^−/−^ and STAT1^+/+^ mice (Figure 8A,B). No significant induction of TNF-α was observed due to the infection with *T. crassiceps* in any group (Figure 8C). We also measured the concentration of the inflammatory cytokine IL-17A. The sera from STAT1^−/−^ infected mice, obtained 8 weeks post-infection, displayed significantly higher amounts of IL-17A compared to STAT1^+/+^ mice (Figure 8D). Thus, STAT1^−/−^ mice are as efficient as STAT1^+/+^ mice in producing cytokines involved in M2 macrophage polarization.

## 3. Discussion

The STAT proteins are a family of transcription factors that have a critical role in the signaling of many cytokines, and they are also involved in the protection against different pathogens [27,28]. Regarding the role of STAT1 in parasitic infections, there is abundant literature indicating the importance of this transcription factor in the defense against intracellular parasitic pathogens and viral infections [29]. In contrast, the role of STAT1 in helminthic infections is understudied. In this context, we previously reported the important role of some of the STAT family proteins during *Taenia crassiceps* infection. The STAT4 protein is involved in resistance against *T. crassiceps* [12], while STAT6-deficient mice display a stronger Th1 immune profile and develop a lower parasite burden than wild-type mice [11]. STAT6 has also been associated with the expansion of alternatively activated macrophages (M2 macrophages) in the chronic stage of this infection, leading to the immunosuppression of Th1 responses [13]. In the present study, we investigated the role of the transcription factor STAT1 during intraperitoneal infection with *T. crassiceps*. Although *T. crassiceps* infection is well-known to polarize the immune response toward a Th2 phenotype, the acute phase of the infection is characterized by an early Th1 response. In the chronic stage, when the parasite burden increases exponentially, peritoneal macrophages polarize to an M2 phenotype. These M2 macrophages are also known to display an impaired response to IFN-γ [13,15], which could be triggered by failed phosphorylation of STAT1 [30]. Here, we first hypothesized that mice lacking STAT1, the transcription factor involved in IFN-γ-mediated responses, would be more susceptible to *T. crassiceps* infection. Instead, we found that STAT1^−/−^ mice were more resistant to this infection. Moreover, STAT1 was necessary to recruit CD11b^+^Ly6C^hi^ cells, a previously described population usually reported as proinflammatory monocytes. Lack of recruitment of these monocytic cells in STAT1^−/−^ mice was associated with the absence of the chemokine receptor CCR2, in line with our data, STAT1 has been implicated in the expression of CCR2 [31]. Importantly, CCR2 mediates cell migration from bone marrow and the recruitment into damaged tissue or inflammation sites of both proinflammatory monocytes and myeloid suppressor cells [32,33]. However, we demonstrated here that the Ly6C^hi^-recruited cells in the early stage of infection with *T. crassiceps* expressed the regulatory molecule PDL-1 and suppressed T-cell proliferation in vitro. Moreover, STAT1^−/−^ mice recruited more neutrophilic Ly6G^+^ cells compared to STAT1^+/+^ mice that were associated with higher levels of IL-17. We propose that STAT1-dependent Ly6C^hi^ cells recruited by *T. crassiceps* infection may be the precursors that give rise to M2 macrophages during the chronic phase of the infection. In support of this idea, during the helminth infection by *Schistosoma mansoni*, it has been reported that Ly6C^hi^ monocytes recruited at the liver become alternatively activated macrophages or M2 macrophages [34,35]. 

Macrophages lacking STAT1 responded to IL-4 in vitro and were able to phosphorylate STAT6 and Akt, meaning that they can be polarized to an M2 phenotype. However, the decreased number of M2 macrophages in STAT1^−/−^ mice suggests that other mechanisms may be involved in this phenomenon. We hypothesize that CD11b^+^Ly6C^hi^CCR2^+^ cells recruited during *T. crassiceps* infection maturate to M2 macrophages in the chronic stage of the infection. Therefore, mice with a deficient recruitment of Ly6C^hi^ cells did not fully develop M2 macrophages. This work also highlights the importance of innate cells, particularly monocytes and macrophages, as facilitators for the establishment and growth of *T. crassiceps*, as reduced development of suppressor monocytes and M2 macrophages correlated with resistance to the infection. Here, we uncover a novel role for STAT1 by showing that mice lacking this transcription factor do not completely develop M2 macrophages during a helminth infection. These data shed light on the emerging role for STAT1 in infections caused by helminth parasites. Recently, it was reported that *Schistosoma* egg antigens modulate STAT1 signaling in human cells [36], a finding similar to that previously reported for *T. crassiceps* infection; in addition, *T. crassiceps*-excreted/secreted products impaired the phosphorylation of STAT1 in response to IFN-γ in both mouse macrophages and human peripheral blood mononuclear cells [15]. Moreover, the impact of helminthic infections on modulating the STAT1/IFN-γ signaling pathway is reflected in coinfections with viruses, where the presence of helminths reactivates latent viral infections [37].

M2 macrophages are found in the chronic stage of *T. crassiceps* infection and correlate with an increased parasitic burden [13]. Depletion of peritoneal macrophages with clodronate liposomes early in the course of *T. crassiceps* infection restricted the parasitic burden [16]. All these data indicate a detrimental role for M2 macrophages during infection with *T. crassiceps*. Here, our new data confirm the detrimental role of M2 macrophages in this infection, since the lack of recruitment of CD11b^+^Ly6^hi^CCR2^+^PDL-1^+^ monocytes in STAT1^−/−^ mice impairs the development of M2 macrophages at the site of infection and favors the elimination of the parasite. Interestingly, a similar phenomenon was observed in visceral leishmaniasis, where a lack of STAT1 inhibited the recruitment of monocytes and the hosts were relatively more resistant to *L. donovani* infection [18].

In summary, STAT1 is an important transcription factor involved in the response to IFN-γ but it also participates in the recruitment of inflammatory monocytes to the site of the infection and the spleen. In particular, STAT1 is a determinant for the expression of CCR2 in monocytes during acute infection with the helminth *T. crassiceps*, which also highly express Ly6C and PDL-1. Hence, these STAT1-dependent CD11b^+^CCR2^+^Ly6C^hi^PDL-1^+^ monocytes with suppressive activity participate in the full establishment of *T. crassiceps* infection and may give rise to M2 macrophages that favor parasite growth.

## 4. Materials and Methods

### 4.1. Mice

Six- to 8-week-old BALB/c were purchased from Harlan Laboratories (Mexico). STAT1^−/−^ mice with a BALB/c genetic background were kindly donated by Dr. Abhay Satoskar (The Ohio State University, Ohio, USA). All mice were housed in a pathogen-free environment at the FES–Iztacala Universidad Nacional Autónoma de Mexico (UNAM) animal facility according to the Faculty Animal Care and Use Committee and government guidelines (official Mexican regulation NOM-062-ZOO-1999), which are in strict accordance with the recommendations in the Guide for the Care and Use of Laboratory Animals of the National Institutes of Health (USA). The protocol was approved by the Committee on the Ethics of Animal Experiments of the FES–Iztacala (UNAM). The mice were euthanized using a CO_2_ chamber and all efforts were made to minimize pain.

### 4.2. Genotype of STAT1 Knockout Mice

A piece of the tail tissue (0.5 cm) of the mice was placed in Eppendorf tubes, and 500 μL lysis buffer (100 mM NaCl, 25 mM EDTA, 0.5% SDS) pH 8 and 10 µL de proteinase K 20 mg/mL (VWR Life Science) were added. Tissues were homogenized in vortex and incubated at 56 °C overnight or 60 °C for 2 hours. Subsequently, the sample was centrifuged at 12000 rpm and the DNA was precipitated with 500 μL of isopropanol (Sigma, Aldrich, USA); then, it was washed four times with 500 μL of 75% ethanol (Sigma Aldrich, USA), the excess of ethanol was evaporated, and the DNA was hydrated with DEPC water. It was quantified and, finally, PCR was performed using 100 ng of DNA, KAPA Taq (Kapa Biosystems, Woburn, MA, USA) and the primers (STAT1 KO WT reverse, common and mutant reverse) described in Table 1.

### 4.3. Parasites and Infection

Parasite harvest and infection were performed as previously described [11]. Briefly, metacestodes of *T. crassiceps* (ORF strain) were harvested under sterile conditions from the peritoneal cavity of female BALB/c mice 8–10 weeks after infection. The cysticerci were washed 5 times in physiological saline solution (PSS) prior to mouse infection. STAT1^+/+^ and STAT1^−/−^ mice were infected with an i.p. injection of 10 small non-budding cysticerci of *T. crassiceps* suspended in 0.3 mL of PSS. The infected mice were sacrificed at 2 days and 8 weeks post-infection (p.i.) and the parasites harvested from their peritoneal cavity were counted.

### 4.4. ELISA for Specific Antibodies and Cytokines 

Blood was obtained by a small cut in the tail vein and centrifuged to obtain serum. Serum was stored at −70 °C until use. Then, 96-well plates (Nunc, Polysorp) were incubated with 10μg/mL of *Taenia crassiceps* soluble antigen overnight at 4 °C. After blockade, serum from the different groups was adjusted at 1:25 dilution with subsequent double dilutions in PBS. After overnight incubation, anti-mouse IgG1 or anti-mouse IgG2a coupled with HRP (Zymed) was added to the plate. IgG levels were evidenced by adding ABTS/H_2_O_2_. For cytokines, sera from STAT1^+/+^ and STAT1^−/−^ *T. crassiceps*-infected or naïve mice were obtained and ELISA for IL-13, IL-10, TNF-α, and IL-17 was performed according to the manufacturer’s instructions (Peprotech, Mexico).

### 4.5. Cell Preparations and Culture Conditions

Peritoneal exudate cells (PECs) were obtained from the peritoneal cavity of non-infected, acute-infected (2 days), and chronic-infected (8 weeks) mice with *T. crassiceps*. PECs were washed twice with PSS and viable cells were counted using a Neubauer hemocytometer by trypan blue exclusion. The spleen was removed under sterile conditions from naïve and 8-week-infected mice. Single-cell suspensions were prepared by gently teasing apart the spleen in supplemented RPMI. The cells were centrifuged, and the erythrocytes were lysed by resuspending the cells in Boyle’s solution (0.17 M Tris and 0.16 M ammonium chloride). Following 2 washes, the viable cells were counted as before. 

Peritoneal exudate cells and splenocytes were adjusted to 5 × 10^6^ cells/mL in supplemented RPMI with 10% fetal bovine serum, 100 units of penicillin/streptomycin, 2 mM glutamine, 25 mM HEPES, and 1% non-essential amino acids (all from GiBCO, Grand Island NY) and cultured in 6-well plates (Costar). After 2 h at 37 °C and 5% CO_2_, the non-adherent PECs were removed by washing with warm supplemented RPMI medium.

### 4.6. Fluorescence Activated Cell Sorting (FACS)

Spleen cells and PECs were analyzed by FACS at 2 days and 8 weeks post-infection and from non-infected mice. Cells were analyzed for the expression of different markers and were previously gated from a high forward light scatter (FSC)/high side light scatter (SSC) gate. For the assay, 1 × 10^6^ cells were incubated with anti-CD16/CD32 antibody (Biolegend, San Diego, CA, USA) to block non-specific binding. Next, cells were stained with specific anti-F4/80, anti-PD-L1, anti-PD-L2, anti-MR, anti-CD11b, anti-Ly6C, anti-Ly6G (all from Biolegend), and anti-CCR2 (R&D Systems) and incubated for 30 min at 4 **°C**. Cells were washed twice with 1 mL of FACS buffer (1% FBS and 0.5% of sodium azide in PBS). Cell analysis was performed using the FACsAria system and FloJo software.

### 4.7. [^3^H] Thymidine Incorporation Assay 

Splenocytes were seeded at a density of 3 × 10^5^ cells per well in triplicate onto 96-well flat-bottom plates (Costar), stimulated with anti-CD3 (2 μg/ml) antibody (BioLegend)-coated plates, and cultured alone or cocultured with peritoneal exudate cells (from STAT1^+/+^ and STAT1^−/−^) at 1:4 ratio (7.5 × 10^4^) for 72 h, maintained at 37 °C and 5% CO_2_. To measure DNA synthesis, [^3^H]-labelled thymidine (0.5 μCi per well) was added to the wells 18 h prior to reach 72 h in culture. Cells were harvested on a 96-well harvester (Tomtec, Toku, Finland) and then counted using a microβ-plate counter (Trilux, Toku, Finland). Values are represented as CPM from triplicate wells.

### 4.8. Suppression-Assays 

In total, 100,000 splenocytes were stained with Cell Trace Violet™ (ThermoFisher®) following the manufacturer’s instructions. Then, they were incubated in a complete RPMI medium in each well of a 96-well plate (Costar) with anti-CD3 antibody (10 μg/mL). After this, 2.5 × 10^4^ peritoneal cells were added for a final 1:4 dilution in a humidified atmosphere containing 5% CO2 in air at 37 °C. After 72 hours of incubation, cells were harvested, stained for CD4 and CD8 molecules, and immediately analyzed by flow cytometry.

### 4.9. Western Blot Assay

Peritoneal exudate cells from STAT1^+/+^ and STAT1^−/−^ mice infected with *Taenia crassiceps* and controls were incubated for 24 h; subsequently, the adherent cells were washed with culture medium and stimulated with or without IL-4 recombinant (20 ng/mL; Peprotech Mexico) for 20 min. Cells were lysed and then 30 µg of protein was boiled for 5 min and separated by SDS-PAGE using 10% polyacrylamide gel, and transferred to polyvinylidene difluoride (PVDF) membranes (Immobilon-P, Millipore). Membranes were blocked with 5% fat-free milk in PBS for 1 h at room temperature and incubated overnight at 4 °C with the primary antibody. The primary antibodies were recognized (Anti-β-actin Biolegend,1:2000; Anti-AKT Cell Signalling,1:1000; Anti-AKT-P Cell Signalling,1:1000; Anti-STAT6 Santa Cruz,1:200, Anti-STAT6-P Santa Cruz,1:150). Then, the detection step was performed with HRP-coupled anti-rabbit IgG (BioLegend 1:2000). Finally, the detection was done with the Chemiluminescent Substrate Kit (VisiGlo, VWR, AMRESCO) and the bands were visualized with the C-DiGit (LI-COR) equipment.

### 4.10. Polymerase Chain Reaction

Total RNA was extracted from peritoneal exudate cells for TRIZOL (Thermo Fisher Scientific, Waltham, MA, USA) according to the manufacturer’s instructions. We performed inverse transcription using the RevertAid H minus First Strand cDNA Synthesis Kit (Thermo Scientific). The obtained complementary DNA was amplified by polymerase chain reaction using KAPA Taq (Kapa Biosystems, Woburn, MA, USA) and the primer set (Table 1). Amplified products were observed on 1.5% of agarose gel with a DocTM-EZ Gel Documentation System. The relative expression of different genes was analyzed using the ImageJ program and normalized with the β-Actin gene.

### 4.11. Statistical Analysis

The data were analyzed using GraphPad Prism 5 by one-way ANOVA, followed by Tukey´s multiple comparison test. The data are expressed as the mean ± SEM, where the significant differences are *p* ≤ 0.05.

## Figures and Tables

**Table 1:** Sequences of the primers used to determine STAT1 deficiency and evaluate M2 macrophage markers by endpoint PCR.

Gene	Sequence Primers	Temperature °C
β-Actin forward β-Actin reverse	TTTGATGTCACGCACGATTTCC TGTGATGGTGGGAATGGGTCAG	60 °C
Arginase-1 forward Arginase-1 reverse	CAGAAGAATGGAAGAGTCAG CAGATATGCAGGGAGTCACC	54 °C
Ym-1 forward Ym-1 reverse	TCACAGGTCTGGCAATTCTTCTG TTTGTCCTTAGGAGGGCTTCCTC	56 °C
Fizz-1 forward Fizz-1 reverse	GGTCCCAGTGCATATGGATGAGACCATAGA CACCTCTTCACTCGAGGGACAGTTGGCAGC	62 °C
STAT-1 KO WT Reverse STAT-1 KO Common STAT-1 Mutant reverse	CTGATCCAGGCAGGCGTT TAATGTTTCATAGTTGGATATCAT GAGATAATTCACAAAATCAGAGAG	52 °C

## Data Availability

All data are reported in the manuscript.
